# Research on Load Reverse Engineering and Vibration Fatigue Analysis Technology of Rapid Box Wagon

**DOI:** 10.3390/ma15238322

**Published:** 2022-11-23

**Authors:** Ji Fang, Xiangwei Li, Dailin Zhang, Xueli Zhang, Wendong Shao

**Affiliations:** 1 College of Locomotive and Rolling Stock Engineering, Dalian Jiaotong University, Dalian 116028, China; 2 CRRC Qiqihaer Rolling Stock Co., Ltd., Qiqihar 161000, China

**Keywords:** rapid box wagon, load reverse engineering, welded structure, modal structural stress method, vibration fatigue

## Abstract

The overall stiffness and modal frequency of the car body of a rapid box car are reduced by the design of the full-side open movable side door structure. The vibration fatigue performance of the welded structure in this car body needs to be verified. The rigid-flexible coupling model of the rapid box wagon was established first, and the model was verified by modal test data. By the application of the virtual iteration method on this model, the displacement excitation loads of this vehicle were acquired. The effectiveness of the load reverse obtaining technology was verified through the comparison between calculated data and the experimental data. Based on the rigid-flexible coupling model and the load obtained by reverse engineering, the fatigue life of the welded structure in the car body was evaluated through the modal structural stress method. The calculated results show that the car body structure obtains obvious modal vibration, which leads to short fatigue life in several weld lines. According to the application requirements of this wagon, the local improvement scheme was proposed, and the effect of the improvement program was evaluated. In this paper, a new fatigue evaluation technology based on the load reverse method of test data was proposed, which provides a theoretical basis for the structural design and program improvement of railway vehicles.

## 1. Introduction

With the development of China’s e-commerce economy, railway freight wagons with high speed, larger capacity, more convenient loading, and lightweight design are needed. Therefore, the 160 km/h rapid box wagon is being designed in China [1]. In order to meet the needs of convenient loading and unloading, a rapid box wagon adopts a full-side open-body structure. Due to there being no side wall structure, the stiffness of the whole car body is obviously reduced compared with previous freight vehicles. In addition, the running speed of rapid box wagons is obviously improved compared with other freight wagons in the past; therefore, the excitation frequency range of railway load has become wider. Due to structural vibration, the probability of fatigue failure in the weld line is increased. At the same time, the safety requirements are higher, which makes it more difficult to design and analysis of the car body structure. The vibration fatigue analysis technology of welded structures has been studied by domestic and foreign scholars. Wang Jujin et al. obtained the S–N curve equation for fatigue life evaluation of ring weld by linear fitting of fatigue data with the least square method and proposed the S–N curve for fatigue life evaluation of ring welded structure [2]. The finite element model based on the main E-N curve method was used to study the sensitivity of the structural strain method to classify weld material Gao Yidi et al. Then, a correction method for elastic finite element calculation was proposed in [3]. Based on the random vibration theory and modal superposition method, and the master S–N curve method, the modal structural stress method in the time domain and the frequency domain structural stress method in the frequency domain for vibration fatigue life prediction were proposed by Fang Ji [4,5,6]. Wang Tengfei et al. predicted the random vibration fatigue life of bogie frames based on multi-dimensional and multi-support pseudo excitation methods for the track irregularity of rail vehicles in operation [7]. Based on the joint test data of high temperature test rig and vibration numerical simulation, the axial dynamic stress response law of the sample root and neck under different temperatures and different vibration magnitudes was obtained [8]. In order to improve the accuracy of vehicle load identification and vehicle fatigue life prediction, based on the improved vibration system transfer function estimation algorithm, the virtual iterative calculation method was proposed by Yu Yuebin et al. [9]. Based on the Newmark-β numerical simulation method, Fan Yuchuan et al. deduced the iterative solution process of the dynamic response of a nonlinear system under external excitation of the system. This method overcomes the error accumulation shortcoming of non-iterative method [10]. Based on discrete time and dynamic state space equations in the time domain, the load identification method was proposed by Wang Ming-meng et al. [11]. A transient load identification method proposed by Zhou Jian et al. based on the strain response and strain model parameters in [12]. Shahbaznia M et al. proposed a new reliability-based method for damage and load identification of railway bridge structures using finite element model updating in the presence of uncertainty [13]. A whole identification process, based on road measurement realized on the carmaker’s proving ground with instrumented vehicles, was developed and discussed in [14]. Farshad Liaghat proposed an effective iterative inverse procedure for the identification of the distribution of loads that had led to a specific crack propagation path in a fractured component and formulated the inverse problem of load distribution identification as an optimization problem [15]. A load spectrum that approximates the service loads was considered in the fatigue assessment of two weld seams in a differential based on notch stresses at the weld root [16].

The well-known fatigue analysis approaches are nominal stress approaches, structural stress approaches, notch stress approaches, fracture mechanics approaches, etc. [17,18]. Due to the limited test data of standard welded joints, sometimes it is difficult to find appropriate S–N curve data when evaluating non-standard welded joints with the nominal stress method. Although the structural stress method solves this problem of limited S–N curve data, its applications are rarely used in complex multi-body dynamic systems [19,20]. The railway vehicle system is a complex multi-body dynamic system, and the load excitation mainly comes from the track irregularity. We urgently need to reduce the degree of freedom of the dynamic model, at the same time, consider the influence of the structural modal vibration on the fatigue life of the weld line in the car body. Therefore, the modal structural stress method was proposed by the author based on the structural stress method and rigid-flexible coupling dynamic model of the vehicle. Since the modal structural stress method is based on the structural stress method, which makes it retains the original mesh insensitivity property of the structural stress method [21]. At present, the main methods of load acquisition can be classified into direct test methods and indirect test methods. Limited by equipment and test conditions, direct methods are mainly applied to simple structures. Among the indirect testing methods, the virtual iteration method based on inversion of transfer function is more mature and is widely used in the automobile industry. In this paper, the virtual iteration technology was applied in the load reverse engineering of the rail vehicle structure. Through the vibration test of the rapid box wagon, the acceleration of key points in the car body and bogie frame was measured. The displacement excitations were obtained by the reverse engineering method and the rigid-flexible coupling virtual test simulation model, which can be used for dynamic simulation and fatigue life evaluation. The modal vibration response of this car body structure was extracted, and the fatigue life of key weld lines in the car body was evaluated through the modal structural stress method. The technology proposed in this paper can provide technical support for the design and improvement of the car body structure.

## 2. Build the Rigid-Flexible Coupling Dynamic Model of Rapid Box Wagon

**Figure 1:**
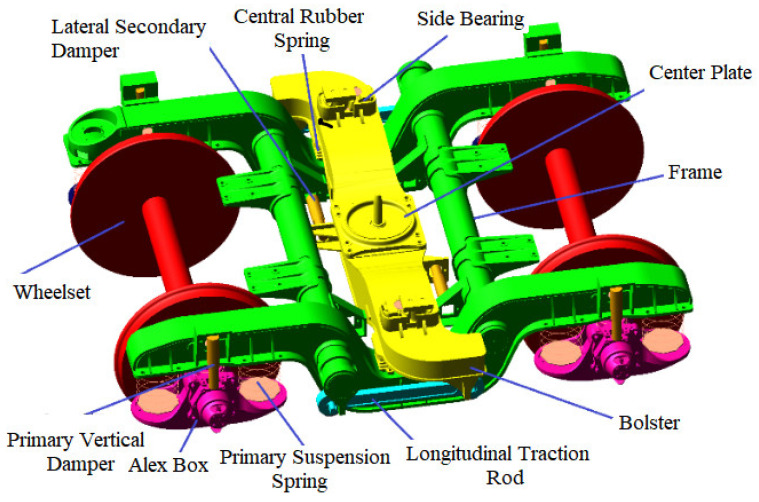
Dynamic model of multi-body bogie system.

**Figure 2:**
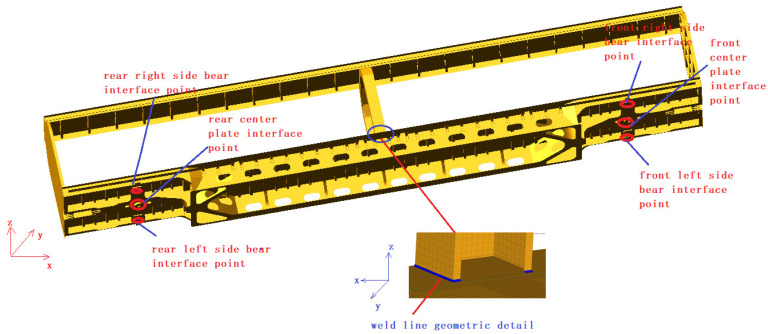
Interface points set of flexible car body model.

The rapid box wagon system consists of two 160 km/h high-speed bogies and a car body. The bogie is mainly composed of wheelsets, axle boxes, primary steel springs, vertical dampers, bogie frames, bolsters, central rubber springs, lateral dampers, side bearings, longitudinal traction rods, etc. A multi-body system licensed simulation software (version 2005) MSC.Adams/View module was used to build the rigid-flexible coupling dynamic model of the rapid box wagon, as shown in Figure 1. Among them, the spring, damper, and side bearing are simulated by force elements (as shown in Table 1 of specific parameters), and other structures are simulated by a rigid body. In order to take the influence of local stress concentration on fatigue life into consideration, the local geometric details of the weld are created in the finite element model of car body structure by ANSYS software (version 14.0). The car body is mainly welded with steel plates of 09CuPCrNi-A and simulated by shell element. The total number of elements is 355,671, and the total number of nodes is 349,109. The improved Craig–Bampton modal set can better fit the deformation of the structure under quasi-static load and the modal vibration under dynamic load at the same time, and it is an orthogonal set that can effectively reduce the degree of freedom of the flexible body. Based on the improved Craig–Bampton modal synthesis method, the flexible body model of the car body was established. The car body is supported by the two bogies. Therefore, the output interfaces of the flexible body were defined at the connection point between the car body and the bogies. The left- and right-side bearings and the center plate in the middle support points were defined as the output interfaces. Six interface points are required for the connection between the front bogie and the rear bogie, and the vehicle body (as shown in Figure 2).

Each interface point has 6 degrees of freedom. Hence the total interface degrees of freedom are 36. In addition, the 52 low-order constraint modes related to internal degrees of freedom were selected. After coordinate transformation, the total number of comprehensive modals extracted from the flexible body is 88.

**Figure 3:**
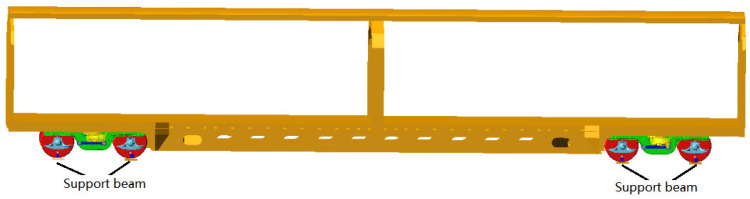
Rigid-flexible coupling dynamic model of rapid box wagon.

The rigid-flexible coupling dynamic model of the rapid box wagon was built by assembling the front and rear bogies with the flexible car body (as shown in Figure 3). The coupling modal shapes and frequencies of the rapid box wagon system can be obtained by solving the eigenvalue of the coupled system dynamic equation, and the main modals are shown in Table 2. Based on the vibration test rig of railway freight car, the modal test of a rapid box wagon was carried out, and the modals of this wagon were acquired by the correlation function matrix and modal parameter identification method [22]. The comparison results between calculated and test data are shown in Table 2.

It can be seen from Table 2 that the maximum error between the calculated and the test frequency of the main operating modals is within 10.2%. The calculated results of the modal vector and frequency of the vehicle operating modal are basically consistent with the test results. It shows that the rigid-flexible coupling model can simulate the modal vibration characteristics of this vehicle very well, which provides the basis for the load reverse calculation and vibration fatigue analysis of this vehicle.

## 3. Vehicle Vibration Test and Key Data Extraction

**Figure 4:**
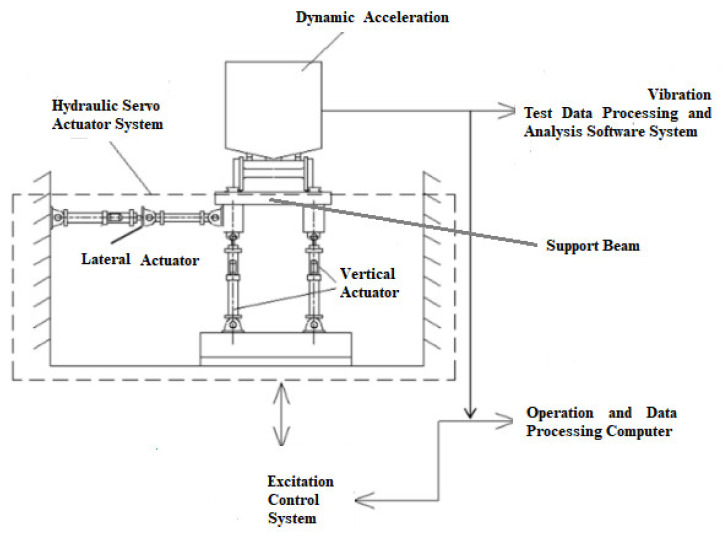
Schematic diagram of the experimental system.

**Figure 5:**
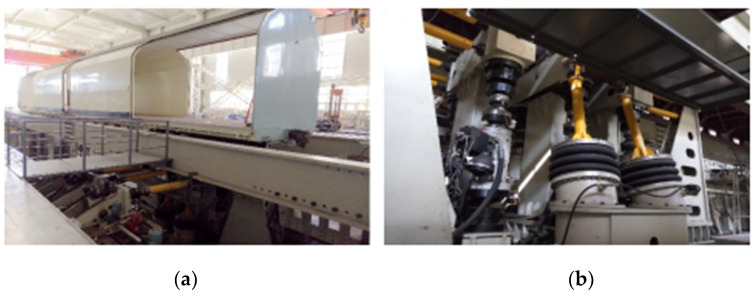
Vibration test of rapid box wagon. (**a**) View of whole vehicle vibration test process; (**b**) view of displacement drive of vibration test rig.

Based on the vibration test of this rapid box wagon, the acceleration data at key points were acquired. The vibration test process of the rapid box wagon is shown in the following Figure 4 and Figure 5. A support beam is provided under each wheelset of the vehicle to support the tested vehicle. Each support beam is excited by two vertical and one lateral electro-hydraulic vibration exciter. The vibration test of this wagon under track irregularity excitations is shown in Figure 5.

**Figure 6:**
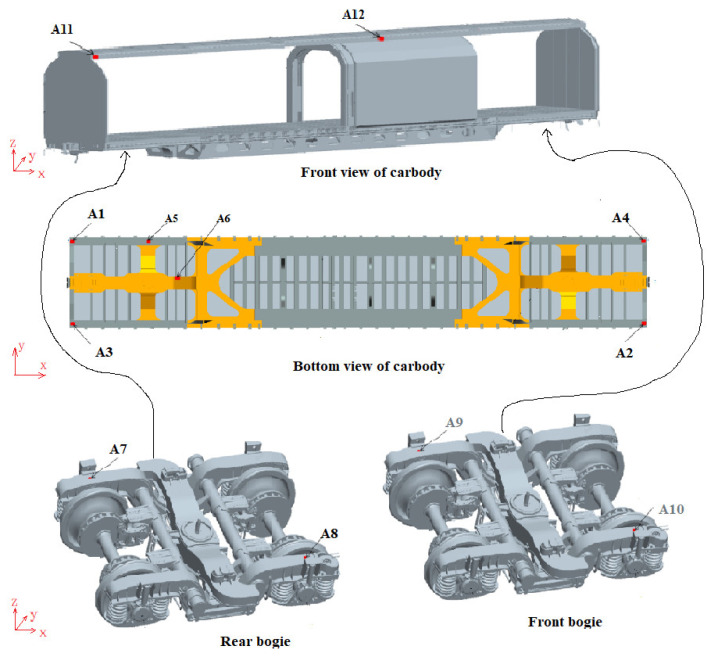
Diagram of acceleration measurement points.

After the acceleration sensor and test equipment were installed, the vibration test of this wagon was carried out. The track irregularity displacement excitations were applied to the wheelset support beams, and the simulated running speed was set to 120 km/h in the operation test. Through the acceleration sensors installed on the car body and bogie frame, the acceleration time history of 12 positions in the car body and frame was measured. Vertical and lateral acceleration sensors were installed at each position, with a total of 24 acceleration measurement channels. The sampling frequency was 512 Hz. The measured signals were pre-processed by burr removal, zero drift removal, noise removal, and filtering; then, the relevant data files were exported. Figure 6 shows the key positions of the acceleration sensor installed in the test. The acceleration sensor brand is PCB353, the sensitivity is ±5%, the measurement range is ±500 g, the frequency range is from 1 to 7000 Hz, and the temperature range is from −54 to 121 °C. Since this type of acceleration sensor is a unidirectional sensor, in order to test the vertical and lateral accelerations, we install both vertical and lateral sensors at each test point.

## 4. Load Reverse Engineering Based on Virtual Iteration of Rigid-Flexible Coupling Model

Based on the rigid-flexible coupling model of the rapid box wagon, the displacement drives were established at the contact points of wheelsets and support beams (as shown in Figure 3). There are two vertical displacement drives and one lateral displacement drive at each wheelset, according to the test. We added 16 channels of acceleration outputs at the car body and 8 channels of acceleration outputs at the bogie frame in the rigid-flexible coupling model according to the test. The total input channels are 12, and the total output channels are 24. Based on the 24 channels of test data, the 12 channels of displacement drive can be obtained through virtual iteration as follow.

In this virtual iteration, the test data of accelerations were taken as the target signal [23,24]. The displacement actuators of the wheelset were taken as the reverse object. Firstly, the white noise displacement actuator signal $u_{noise}$ with an initial input of 0~250 Hz was generated. The initial response signal of the system is $y_{noise}$ through the calculation of a rigid-flexible coupling model through Equation (1).

(1) $$f\left(s\right)=y_{noise}/u_{noise}$$

The inverse transfer relation $f^{-1}(s)$ of the rigid-flexible coupling model can be obtained where s is the complex frequency, and then, the acceleration measurement signal $y_{\;\mathrm{desired}}\left(s\right)$ is the target signal. Based on the inverse transfer function $f^{-1}(s)$, a new displacement excitation signal $u_{1}(s)$ can be calculated by Equation (2).

(2) $$u_{1}(s)=f^{-1}\left(s\right)y_{desired}(s)$$

A complex rigid-flexible coupling system is usually nonlinear. There are certain deviations from the transfer function obtained by local linearization of Equation (1). Therefore, the iterative response $y_{k}(s)$ is not equal to $y_{\mathrm{desired}}\left(s\right)$. Through repeated iteration of Equation (3), the error between the calculated response and the target signal will be continuously smaller.

(3) $$u_{k+1}\left(s\right)=u_{k}\left(s\right)+f^{-1}\left(s\right)\left(y_{desired}(s)-y_{k}(s)\right)$$

where k is the number of iterations. The response of $y_{k}(s)$ to the rigid-flexible coupling model under the displacement excitation load $u_{k}(s)$ by k wheelsets. When the model is a multi-input multi-output system, the transfer function f(s) can be expressed in the form of [F(s)] matrix. In this case, the output channel quantity of the system must be greater than the input channel quantity.

(4) $$u_{k+1}(s)={\left[F^{T}(s)F(s)\right]}^{-1}\left[F^{T}(s)F(s)\right]u(s)={\left[F^{T}(s)F(s)\right]}^{-1}F^{T}(s)y(s)$$

**Figure 7:**
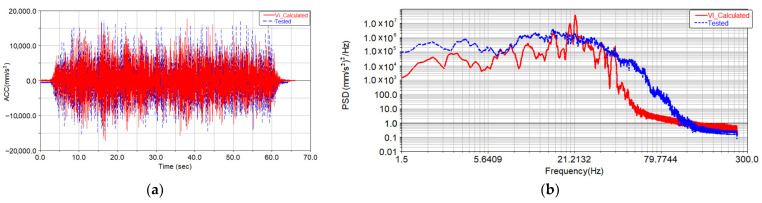
Comparison of vertical acceleration and power spectral density of A11 measuring point. (**a**) Comparison of tested and calculated acceleration; (**b**) power spectral density of tested and calculated acceleration.

**Figure 8:**
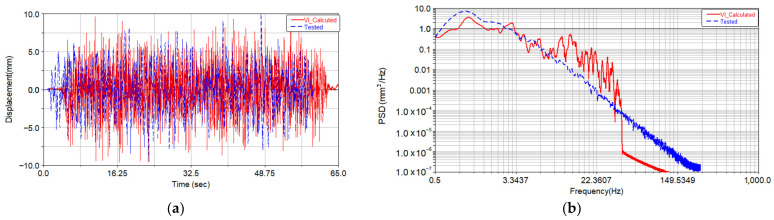
Comparison of time history and power spectral density of lateral displacement actuator. (**a**) Comparison of tested and calculated acceleration; (**b**) power spectral density of tested and calculated acceleration.

In order to reduce the amount of calculation and improve the convergence speed of iterative, the relative root mean square (RMS) maximum error between the response signal and the target signal was set to 5%. Generally, when the maximum error of RMS is set to 5%, satisfactory results will be obtained with fewer iteration steps, according to [25]. Based on the above virtual iteration method, the final displacement driving inputs were obtained after 18 iterations. The acceleration of the car body measuring point A11 was compared to the calculated acceleration from iteration, as shown in Figure 7. The comparison between the calculated lateral displacement actuator of track irregularity in the dynamic model and the displacement actuator applied in the test is shown in Figure 8.

Compared with Figure 7, it can be seen that the amplitude of vertical acceleration from the virtual iteration method is basically consistent with the measured target signal, and their characteristic distributions in the frequency domain are basically consistent. It can be seen from Figure 8 that the amplitude of the displacement actuator obtained through the virtual iteration process is basically consistent with the test result, and their energy distribution in the frequency domain is basically consistent.

## 5. Rigid-Flexible Coupling Simulation and Fatigue Life Prediction of Rapid Box Wagon

### 5.1. Synthesis Modal of Flex Body

The method of flexible body definition in the rigid-flexible coupling dynamic model is the modal synthesis method. Craig–Bampton modal synthesis method is the most representative method [26]. First, the structural degrees of freedom *u* are divided into internal degrees of freedom *u_I_* and interface degrees of freedom *u_B_*. The location of the structure interacting with the outside rigid body is called the interface, such as the position of load application and constraints. Then according to Equation (5) the fixed-interface modals and constraint modals can be calculated. Using the modal vector matrix Equation (6), the physical coordinate can be expressed by the combination of fixed-interface modals and constraint modals. {ξ} is the modal coordinates and can be divided into interface degrees related $\left\{\xi_{C}\right\}$ and internal degrees related $\left\{\xi_{N}\right\}$.

(5) $$\left[\begin{array}{cc} M_{II} & M_{IB}\\ M_{BI} & M_{BB} \end{array}\right]\left\{\begin{array}{c} {\ddot{u}}_{I}\\ {\ddot{u}}_{B} \end{array}\right\}+\left[\begin{array}{cc} K_{II} & K_{IB}\\ K_{BI} & K_{BB} \end{array}\right]\left\{\begin{array}{c} u_{I}\\ u_{B} \end{array}\right\}=\left\{\begin{array}{c} 0\\ F_{B} \end{array}\right\}$$

(6) $$u=\left[\Phi \right]\left\{\xi \right\}=\left[\begin{array}{cc} I & 0\\ \Phi_{IC} & \Phi_{IN} \end{array}\right]\left\{\begin{array}{c} \xi_{C}\\ \xi_{N} \end{array}\right\}$$

In Equation (5), $\left[M_{II}\right]$,$\left[M_{IB}\right]$,$\left[M_{BI}\right]$,$\left[M_{BB}\right]$ are the divided block mass matrixes according to internal degrees and boundary degrees of freedom. $\left[K_{II}\right]$,$\left[K_{IB}\right]$,$\left[K_{BI}\right]$,$\left[K_{BB}\right]$ are the divided block stiffness matrixes according to internal degrees and boundary degrees of freedom. $\left\{F_{B}\right\}$ is the interface force vector. [I] is the unit matrix, and $\left[\Phi_{IC}\right]$ is the internal degree freedom vector of the constrained modal, and $\left[\Phi_{IC}\right]$ is the fixed interface degree freedom of the main modal set. Craig–Bampton modal synthesis method can easily acquire the required structural deformation, but the Craig–Bampton modal base is not a set of orthogonal basis. For the purpose of modals from the Craig–Bampton modal synthesis method can better serve the solution of dynamic equations, Modal orthogonalization for Craig–Bampton modal is required. Solving the undamped free vibration equation of the transformed Equation (7), then the new eigenvalues and eigenvectors matrix *N* can be acquired. Craig–Bampton modal can be orthogonalized by the transformation matrix *N*, then the physical coordinates can be expressed by modal $\phi_{j}^{*}$. The new modal base $\left[\Phi^{*}\right]$ is a set of orthogonal basis. $q_{j}$ is the jth modal coordinate, and R is the total number of selected modal.

(7) $${\left[\Phi \right]}^{T}\left[M\right]\left[\Phi \right]\left\{\ddot{q}\right\}+{\left[\Phi \right]}^{T}\left[K\right]\left[\Phi \right]\left\{q\right\}=0$$

(8) $$u=\sum_{j=1}^{R}\phi_{j}\xi_{j}=\sum_{j=1}^{R}\phi_{j}Nq_{j}=\sum_{j=1}^{R}\phi_{j}^{*}q_{j}$$

Normally, the specific values of the eigenvectors are uncertain. In order to obtain quantitative eigenvectors, we need modal regularization. Which can be normalized by mass matrix Equation (9) is one of the most commonly used methods of regularization.

(9) $${\left[\Phi^{*}\right]}^{T}M\left[\Phi^{*}\right]=\left[I\right]$$

### 5.2. Definition of Modal Structural Stress

**Figure 9:**
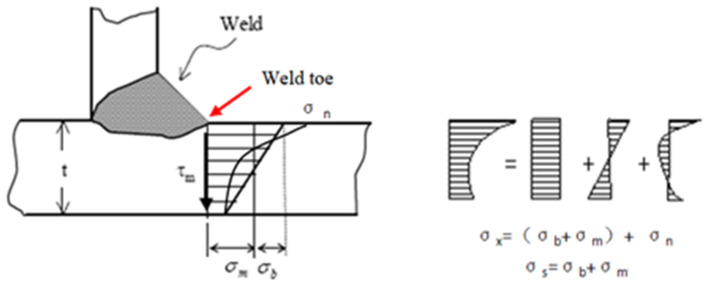
Stress distribution at the weld toe.

Structural stress in weld toe $\sigma_{x}$ equal the sum of membrane stress $\sigma_{m}$, bending stress $\sigma_{b}$, and nonlinear peak stress $\sigma_{n}$, according to [17]. Nonlinear peak stress is self-balanced notch stress (as shown in Figure 9).

Since the definition of structural stress is based on node force, the corresponding modal node force of each modal can be obtained by using the following Equation (10) according to the modal vector $\left[\Phi^{*}\right]$.

(10) $$\left\{F_{j}\right\}=BK^{e}B^{-1}\left\{\phi_{j}^{e}\right\}$$

$K^{e}$ is the element stiffness matrix in local coordinates.
$\phi_{j}^{e}$ is the *j*-order modal vector corresponding to the nodes in element *e*.
B is the transform matrix from the local coordinate of the element to the global coordinate.

**Figure 10:**
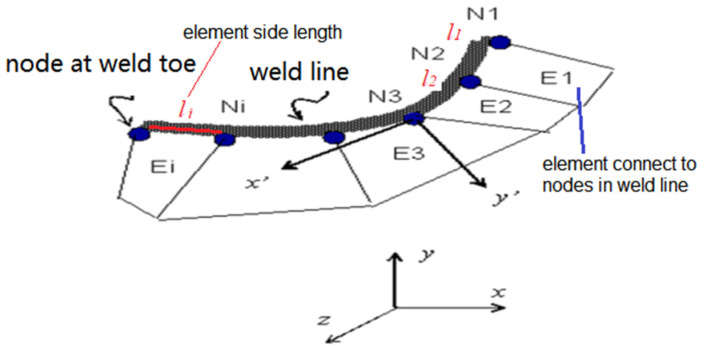
Node force of weld line in local coordinate.

Based on the definition of structural stress, the node force matrix at the weld toe under the global coordinate system needs to be transformed to the local coordinate of the weld line (as shown in Figure 10). Then the node force is converted to the node line force by equivalent matrix Equation (12) according to the principle of work equivalence. $l_{i}$ is the side length of the relevant element, and [L] is the transform matrix.

(11) $$L=\left[\begin{array}{cccccc} \frac{l_{1}}{3} & \frac{l_{1}}{6} & 0 & 0 & \cdots & 0\\ \frac{l_{1}}{6} & \frac{l_{1}+l_{2}}{3} & \frac{l_{2}}{6} & 0 & \cdots & 0\\ 0 & \frac{l_{2}}{6} & \frac{l_{2}+l_{3}}{3} & \frac{l_{3}}{6} & 0 & 0\\ 0 & \ddots & \ddots & \ddots & \ddots & 0\\ 0 & \ddots & \ddots & \ddots & \frac{l_{n-2}+l_{n-1}}{3} & \frac{l_{n-1}}{6}\\ 0 & \cdots & \cdots & \cdots & \frac{l_{n-1}}{6} & \frac{l_{n}}{3} \end{array}\right]$$

(12) $$\{\begin{array}{c} {\left\{f_{j}\right\}}^{T}={\left\{{F^{\prime }}_{j}\right\}}^{T}L^{-1}\\ {\left\{m_{j}\right\}}^{T}={\left\{{M^{\prime }}_{j}\right\}}^{T}L^{-1} \end{array}$$

(13) $$\left\{\sigma_{}^{j}\right\}=\frac{\left\{f^{j}\right\}}{d}+6\frac{\left\{m^{j}\right\}}{d^{2}}$$

In Equation (12), ${\left\{f_{j}\right\}}^{T}$ and ${\left\{M_{j}\right\}}^{T}$ are the node line force and line moment corresponding to the *j*th order modal. ${\left\{F_{j}\right\}}^{T}$ and ${\left\{F_{j}\right\}}^{T}$ are the node force and moment corresponding to the *j*th order modal. The structural stress $\left\{\sigma^{j}\right\}$ in Equation (13) is calculated based on a modal vector, so it can be defined as modal structural normal stress. d is the plate thickness. After the time history of modal coordinates is obtained from the calculation of the rigid, flexible coupling model, the vibration response of structural stress can be calculated by the modal superposition method.

### 5.3. Dynamic Structural Stress Calculation and Fatigue Life Assessment of Key Weld Lines in Car Body

The coupling relationship between the flexible car body and bogie was built through the constraint equation at the interface points of the flexible body. The constraint conditions were introduced into the system equation by the Lagrange multiplier method. The dynamic equation of the rigid-flexible coupling system can be expressed as follows:

(14) $$\begin{array}{c} \frac{d}{dt}\left(\frac{\partial L}{\partial \dot{\xi }}\right)-\frac{\partial L}{\partial \xi }+\frac{\partial F}{\partial \dot{\xi }}+{\left[\frac{\partial \Psi }{\partial \xi }\right]}^{T}\lambda -Q=0\\ \begin{array}{cccc} & & & \begin{array}{cccc} & & \Psi (\xi ,t)=0 & \end{array} \end{array} \end{array}\}$$

In Equation (14), Ψ(ξ,t) is a constraint equation. ξ is a generalized coordinate, including displacement coordinate X, Euler angle coordinate ω, and modal coordinate q. Q is the generalized force. L is Lagrange function. λ is an undetermined factor. F is the dissipation function. The response of the dynamic equation can be solved by the New-mark integral method.

Finally, the structural stress results $\left\{\sigma_{s}(t)\right\}$ under physical coordinates were obtained by modal superposition method. The time history of structural normal stress at the weld line under physical coordinates can be obtained by modal superposition of the following formula. In Equation (15), $q_{j}\left(t\right)$ is the *j*th order modal coordinate time history.

(15) $$\left\{\sigma_{s}^{}(t)\right\}=\sum_{j=1}^{R}\left\{\sigma_{}^{j}\right\}q_{j}(t)$$

(16) $$\Delta S_{S}=\frac{\Delta \sigma_{S}}{d^{(2-m)/2m}\cdot I{\left(r\right)}^{1/m}}$$

In Equation (16), I(r) is a dimensionless function of the bending ratio r ($r=\Delta \sigma_{b}/\Delta \sigma_{s}$). m = 3.6 according to the ASME index [27]. d is the plate thickness. According to the rainflow statistics, the equivalent structural stress variation range $\Delta S_{S}^{i}$ and the corresponding cycles $n_{i}$ of different grades can be obtained. Combined with Miner linear damage accumulation, the fatigue life *N* calculation equation can be expressed as follows:

(17) $$N=\frac{1}{\sum_{i=1}^{k}{\left(\frac{C_{d}}{\Delta S_{S}^{i}}\right)}^{\frac{1}{h}}\cdot n_{i}}$$

The vibration fatigue life of welded structure under time domain loads can be calculated through Equation (17) based on the Master S–N curve. Where $C_{d}$ and h are the mainly related parameters of the S–N curve. It can be referred to as ASME standard [27] according to specific requirements (In the product design stage, the −2σ Master S–N curve is usually suitable). k. is the number of divided grades of equivalent structural stress range, *k* = 64 is widely used and is accepted in this paper.

**Figure 11:**
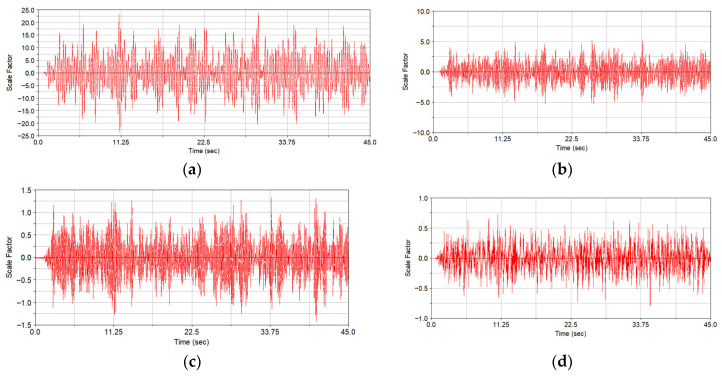
Time history of the first four structural modal coordinates; (**a**) time history of the 7th modal coordinate; (**b**) time history of the 8th modal coordinate; (**c**) time history of the 9th modal coordinate; (**d**) time history of the 10th modal coordinate.

**Figure 12:**
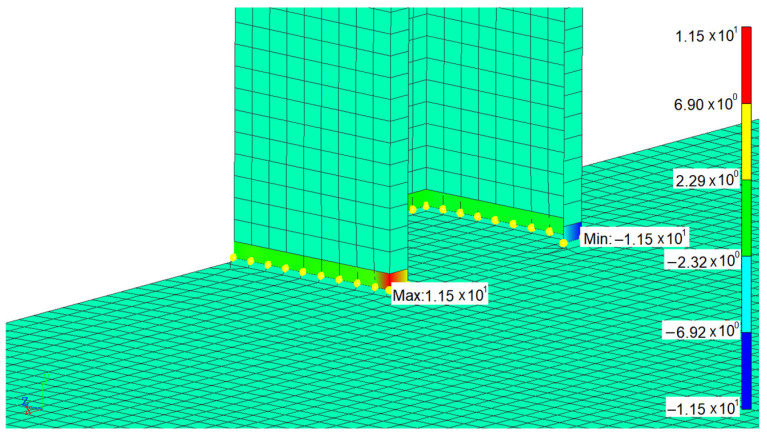
Stress distribution of the 7th modal structure in weld line 1.

The modal structural stress method was used to calculate the variation of structural stress of the weld line in the time domain. Compared with the direct time-domain structural stress-solving method, it can greatly reduce the amount of calculation. The advantages of the modal structural stress method compared with the quasi-static finite element method were verified in reference [5]. The vibration fatigue life prediction technology of welded structure based on the modal structural stress method has the following two advantages: (1) The mesh insensitivity of the structural stress method is maintained. (2) At the same time, the influence of load frequency on the vibration fatigue life of welded structures is considered according to [5]. The excitation load of track irregularity displacement obtained by virtual iteration reverse engineering was applied to the rigid-flexible coupling dynamic model. The simulation speed was set to 160 km/h, and the 45 s of dynamic simulation calculation was completed. Then, the flexible body modal coordinate time history can be extracted from the dynamics calculation result. The first six order modals are rigid body modals, which were ignored in the simulation process. From the 7th order above, it is the time history of modal coordinates related to the vibration of the structure itself. Figure 11 shows the coordinate time history of the 7th–10th-order structural modals. The modal structure distribution in weld line 1 corresponding to the 7th modal is shown in Figure 12 as follows.

**Figure 13:**
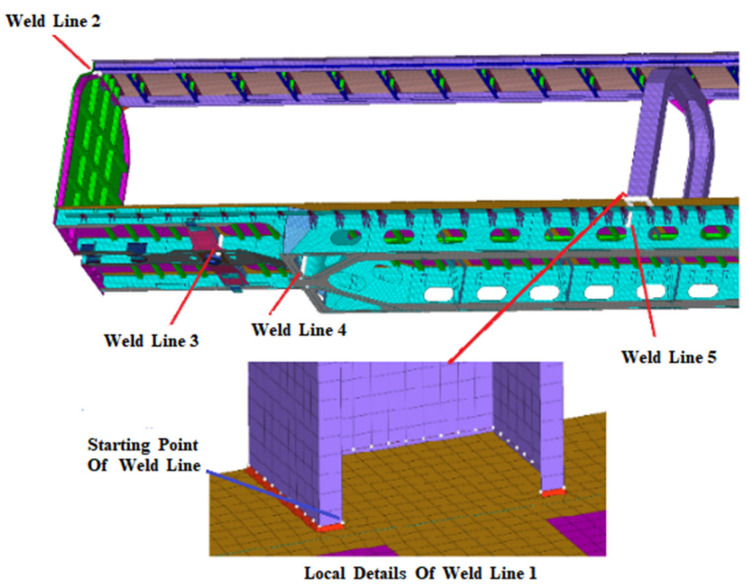
Definition of weld line.

There are too many weld lines in the car body structure; five representative key weld lines with relatively low life were extracted from the car body to discuss in this paper. The position and definition of five selected weld lines are shown in Figure 13.

**Figure 14:**
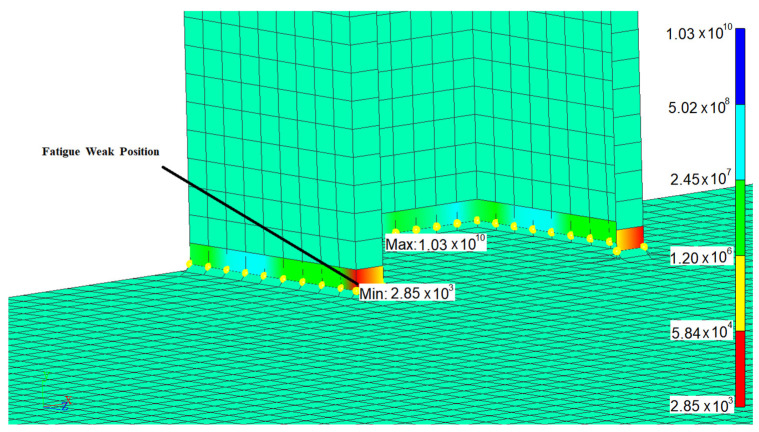
Fatigue life distribution of weld line 1.

**Figure 15:**
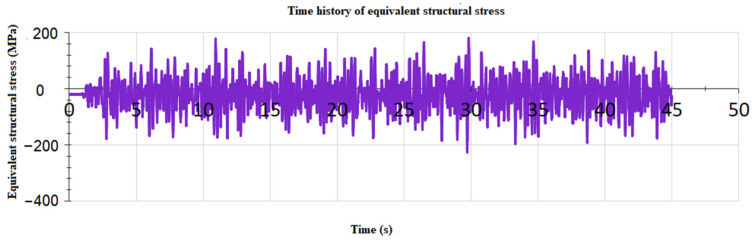
Time history of equivalent structural stress at fatigue weak position in weld line 1.

Each weld line was calculated separately. Firstly, the time history of structural stress was obtained by superimposing the modal structural stress and modal coordinate time history. After rainflow counting, the equivalent structural stress variation range of each node in the weld line can be obtained. The fatigue life of each node in weld lines was calculated based on the Master S–N curve, where $C_{d}$ is 13,876.4 and h is 0.32. The fatigue life distribution of weld line 1 can be seen in Figure 14. The equivalent structural stress time history at low life point is shown in Figure 15. The fatigue life summary of key weld lines is displayed in Table 3.

## 6. Life Evaluation of Proposed Local Improvement Scheme of Car Body Structure

**Figure 16:**
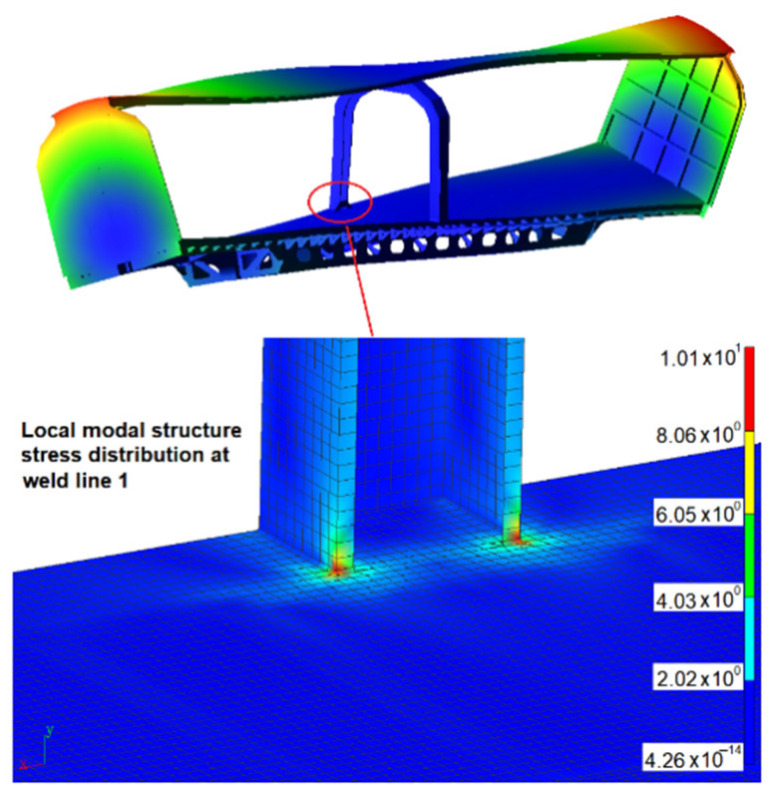
The 7th modal shape and modal stress (4.56 Hz).

**Figure 17:**
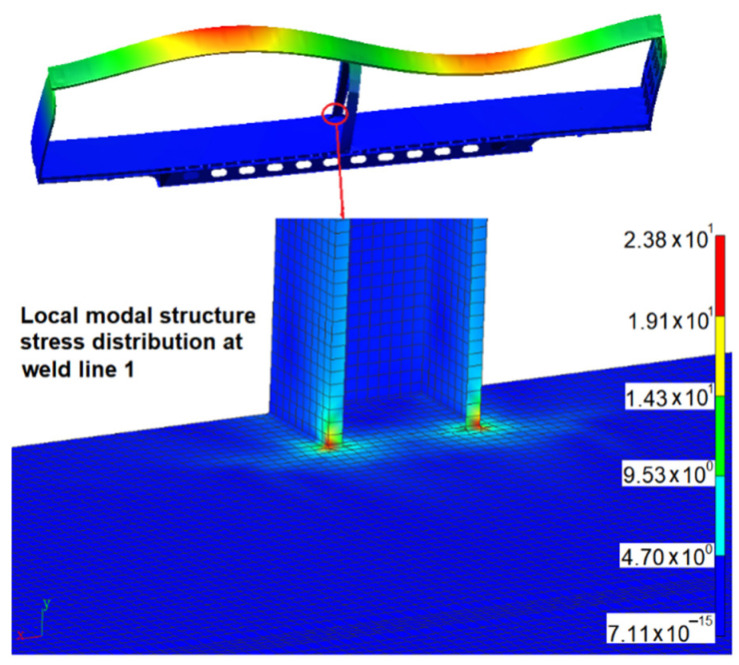
The 8th modal shape and modal stress (5.86 Hz).

**Figure 18:**
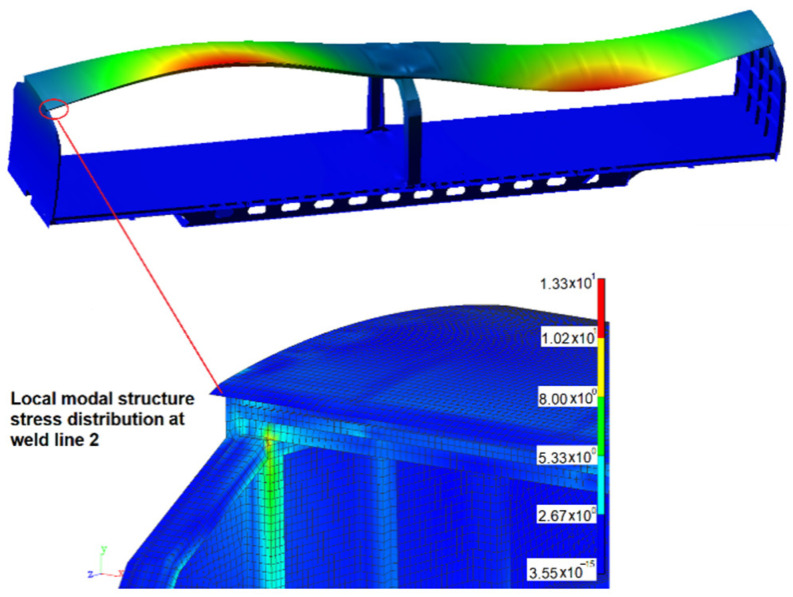
The 9th modal shape and modal stress (6.37 Hz).

It can be seen from Table 3 that weld lines 1 and 2 obtain more short life, and the minimum fatigue life is 5700 km. The main modals related to weld line 1 are the 7th and 8th modals, with frequencies of 4.56 Hz and 5.86 Hz, respectively. The modal shapes and modal stresses are shown in Figure 16 and Figure 17 below. The main modal related to weld line 2 is the 9th modal, with frequencies of 6.37 Hz, and its modal shape and modal stress are shown in Figure 18.

**Figure 19:**
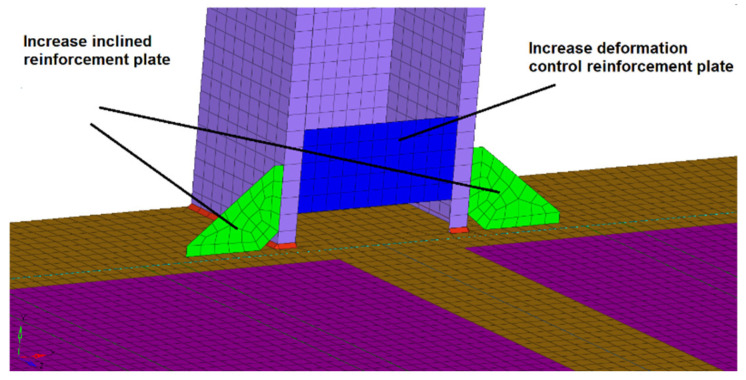
Local improvement scheme of weld line 1.

**Figure 20:**
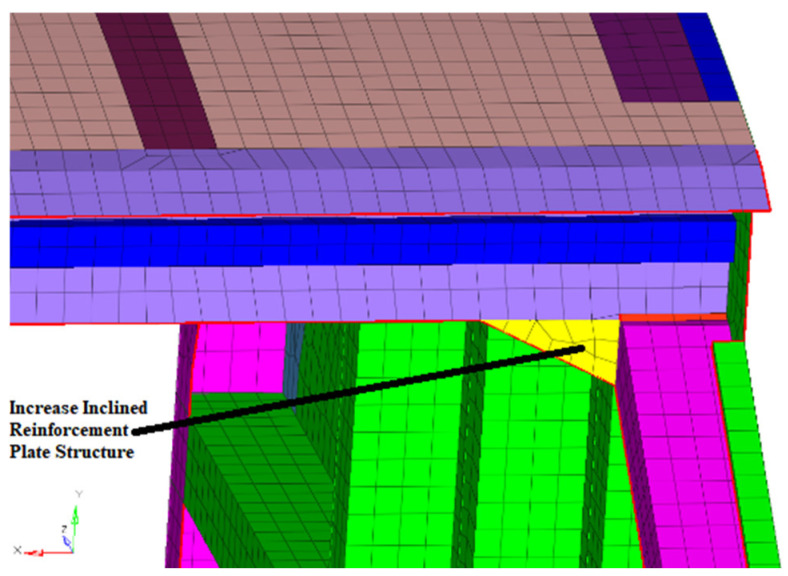
Local improvement scheme of weld line 2.

The 7th modal shape is the torsional mode of the car body. The 8th and 9th modals are mainly bending modals at the top of the car. These modal shapes belong to the vibration of the overall car body. If you want to change this modal, it is necessary to change the overall structural form of the car body. As a matter of fact, it is difficult to change the overall structural form. Therefore, it is suitable to do some research on small change schemes. We focused on how to reduce the local stress concentration. The key method of reducing the local stress concentration in the weld line is to make its local stiffness compatible or reduce the load transmitted by the weld line. The reinforcement plate support structures were added at the local position where the fatigue life is short so as to relieve the local stress concentration and improve the fatigue life. The local improvement scheme for weld line 1 is shown in Figure 19. The local improvement scheme for weld line 2 is shown in Figure 20.

It can be seen from Table 4 fatigue life of weld lines in the local improvement scheme is obviously improved. However, the fatigue life of weld lines 1 and 2 is still relatively short due to weld lines 1 and 2 being at the key points of modal vibration. The newly added weld lines in the local improvement may also have a risk of fatigue failure. Therefore, it is not easy to solve the problem through local structure improvement when the weld line is placed in the key position of the modal shape. It is necessary to optimize the overall structure and avoid setting the weld line in the key position of the modal shape. In addition, the effects of residual stresses as the result of the welding process on the weld joints are ignored or to be assumed that residual stresses in the weld lines are stress relieved. The existence of residual stresses can reduce the fatigue life of the weld joints.

## 7. Conclusions

(1) By comparing the calculated operating modal results of the rigid-flexible coupling dynamic model and test, the error between the calculated and tested modal frequencies is smaller than 10.2%. The calculated results of the modal shape and frequency of the rigid-flexible coupling model of the rapid box wagon are very close to the test results. It shows that the rigid-flexible coupling model built in this paper can simulate the modal vibration characteristics of a rapid box wagon very well, which provides the basis for the load reverse calculation and vibration fatigue evaluation of this vehicle.
(2) The 24 channels of acceleration-tested data output from the vibration test rig were taken as the target parameters. The virtual iteration method was applied to reverse calculate the displacement actuator of the wheelset. It can be seen from the comparison results that the displacement actuators obtained by the reverse calculation of the rigid-flexible coupling model are basically consistent with the loads applied in the test. It shows that the virtual iterative load reverse engineering technology based on the rigid-flexible coupling model proposed in this paper can effectively serve the load reverse engineering of railway vehicles.
(3) Through the fatigue life evaluation of weld lines in the car body structure, it can be seen that the stiffness distribution of the rapid box wagon car body structure is inharmonious. The fatigue life of related weld lines is very short due to the influence of some low-order modal vibration. The local improvement schemes proposed for the car body are effective, and the fatigue life is significantly improved. However, the life in those weld lines is still relatively short due to those weld lines being in the key positions of modal vibration. The fatigue life prediction of weld lines did not consider the effect of residual stresses in the weld lines and other factors that may affect the fatigue life of weld lines in the rapid box wagon car body. Therefore, it is not easy to solve the problem by improving the local structure of the weld line at the key position of the modal shape. It is necessary to optimize the overall structure to solve this problem completely.
(4) The influence of modal vibration on the fatigue life of welded structures can be considered in the modal structural stress method. At the same time, it can identify the key modals that lead to the short fatigue life of weld lines and provide technical support for the design and improvement of the railway vehicle structure. This method can also be applied to the anti-fatigue design of the welded structure in other industries.

## Figures and Tables

**Table 1:** Basic parameters of rapid box wagon dynamic (measured).

Title	Numerical Value	Unit
Gauge	1.435	m
Center distance of shaft diameter	2.0	m
Rolling circle radius	0.457	m
Maximum axle weight	18	t
Maximum Running Speed	160	km/h
Mass of bogie frame	1.9	t
Mass of bolster	0.8	t
Mass of wheelset	1.8	t
Vertical stiffness of side bear	1.70 × 10^3^	kN/m
Longitudinal connection stiffness of longitudinal traction rod	8.42 × 10^4^	kN/m
Lateral deflection angle stiffness of longitudinal traction rod	47.69	N.m/deg
Vertical damping coefficient of each axle box	7.3	kN.s/m
Damping coefficient of each lateral damper	5.7	kN.s/m
Lateral elastic stop stiffness of bolster	1.47 × 10^3^	kN/m
Longitudinal stiffness of axle box spring	4.81 × 10^3^	kN/m
Lateral stiffness of axle box spring	2.39 × 10^3^	kN/m
vertical stiffness of axle box spring	1.19 × 10^3^	kN/m
Lateral equivalent stiffness of central rubber spring	3.20 × 10^2^	kN/m
Vertical equivalent stiffness of f central rubber spring	7.10 × 10^2^	kN/m

**Table 2:** Comparison of vehicle operating modals.

Modal	Modal Vector	Description	Calculated Frequency (Hz)	Test Frequency (Hz)	Error (%)
1	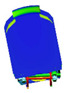	Side roll and center swing of car body	0.99	0.97	2.1
2	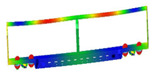	Car body ups and downs	2.98	3.06	−2.6
3	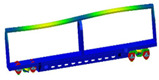	Car body nod	3.62	3.49	3.7
4	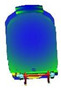	Car body sway	2.69	2.44	10.2
5	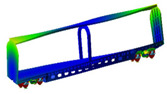	Car body torsion	4.87	5.41	−9.9

**Table 3:** Fatigue life summary of key weld lines.

Weld Line ID	Maximum Equivalent Structural Stress Range (MPa)	Cumulative Damage	Total Life (Ten Thousand Kilometers)
1	406	3.51 × 10^−4^	0.57
2	146	1.53 × 10^−5^	13.08
3	38.3	2.43 × 10^−7^	824
4	32.4	2.09 × 10^−7^	958
5	22.9	4.40 × 10^−8^	4340

**Table 4:** Fatigue life comparison of local improvement scheme.

Weld Line ID	Weld Line Position	Weld Line Life of Original Structure (Ten Thousand Kilometers)	Weld Line Life of Improved Improvement Scheme (Ten Thousand Kilometers)	Increased Life Coefficient
1	Welded place between central column and floor	0.57	25	43.8
2	Joint between ceiling and end wall	13.8	228	16.5

## Data Availability

Not applicable.
